# Modulation of HBV replication by microRNA-15b through targeting hepatocyte nuclear factor 1α

**DOI:** 10.1093/nar/gku260

**Published:** 2014-04-04

**Authors:** Xiaopeng Dai, Wei Zhang, Hongfei Zhang, Shihui Sun, Hong Yu, Yan Guo, Zhihua Kou, Guangyu Zhao, Lanying Du, Shibo Jiang, Jianying Zhang, Junfeng Li, Yusen Zhou

**Affiliations:** 1 State Key Laboratory of Pathogen and Biosecurity, Beijing Institute of Microbiology and Epidemiology, Beijing 100071, China; 2 Henan Key Laboratory of Tumor Epidemiology, College of Public Health, Zhengzhou University, Zhengzhou, Henan 450052, China; 3 Laboratory of Viral immunology, Lindsley F. Kimball Research Institute, New York Blood Center, New York, NY 10065, USA; 4 Department of Biological Sciences, The University of Texas at El Paso, El Paso, TX 79968, USA

## Abstract

Hepatitis B virus (HBV) infection remains a major health problem worldwide. The role played by microRNAs (miRNAs) in HBV replication and pathogenesis is being increasingly recognized. In this study, we found that miR-15b, an important miRNA during HBV infection and hepatocellular carcinoma development, directly binds hepatocyte nuclear factor 1α (HNF1α) mRNA, a negative regulator of HBV Enhancer I, to attenuate HNF1α expression, resulting in transactivation of HBV Enhancer I, in turn causing the enhancement of HBV replication and expression of HBV antigens, including HBx protein, finally leading to the down-regulated expression of miR-15b in both cell lines and mice in a long cascade of events. Our research showed that miR-15b promotes HBV replication by augmenting HBV Enhancer I activity via direct targeting HNF1α, while HBV replication and antigens expression, particularly the HBx protein, then repress the expression of miR-15b. The reciprocal regulation between miR-15b and HBV controls the level of HBV replication and might play a role in persistent HBV infection. This work adds to the body of knowledge concerning the complex interactions between HBV and host miRNAs.

## INTRODUCTION

MicroRNAs (miRNAs) are a family of endogenous conserved noncoding RNAs, about 21–25 nucleotides (nts) in length, which can regulate gene expression at post-translational level by incomplete or complete complementary to 3’-untranslated region (3’-UTR) of the target transcripts (1). The miRNAs play pivotal roles in diverse biological processes including cell development, differentiation, apoptosis, metabolism, stress response and virus infection. The miRNA-associated pathway plays an important role in virus–host interactions. It has been well demonstrated that viruses can either activate or repress the expression of specific cellular miRNAs (2). The disruption of this process can perturb the ability of viruses to replicate normally. In addition, it is currently evident that virally encoded miRNAs play a key role in inhibiting antiviral innate immune responses (3). In order to eliminate viral infections in host cells, cellular miRNA can be directly involved in the process of antiviral immune response by inhibiting or promoting viral replication. Meanwhile, some viruses can encode miRNA that may not only regulate viral gene expression to benefit for their life cycle and maintain latency but also affect host gene expression to accommodate life cycle (2). It has been shown that the miRNA target sequences in the viral populations are conservative, which can help us to evaluate the biological significance of the antiviral effects and then to develop miRNA-based strategies for antiviral intervention (4). Therefore, the continuous study on the role of miRNA in host–virus interaction is of great significance for understanding the pathogenesis and biology of viruses.

Hepatitis B virus (HBV) can cause either acute or chronic hepatitis B in infected individuals, and it has been considered as a high risk factor for chronic liver cirrhosis (LC) and hepatocellular carcinoma (HCC) (5). There are over 400 million HBV carriers worldwide, of which more than 30% are Chinese, and the numbers are still rising (6). Thus, understanding the mechanism underlying acute or chronic HBV infection and LC, as well as HCC development, is of great importance for the management of HBV infection.

A number of studies have been done to identify differentially expressed miRNAs in HCC tissues versus normal liver tissues (7–11) or HBV-infected cells versus control cells (12,13). Among the miRNAs identified, miR-15b is frequently reported to be up-regulated in HCC (14). Interestingly, in cultivated cells, miR-15b has been reported to be down-regulated in acute HBV infection (13). In comparing HepG2, HepG2.2.15 (stable cell line with low HBV replication) and HepAd38 (stable cell line with higher HBV replication than HepG2.2.15) cells (15), we observed that the expression of miR-15b decreased as HBV level increased and that miR-15b was the only miRNA to cause a significant increase in HBeAg expression when differentially expressed miRNAs were transfected into HBV-expressing cells (data not shown).

Several studies have demonstrated that miR-15b may be a potential HCC marker (16) and that miR-15b up-regulation appears to be negatively associated with HCC relapse (14). HBV infection is very common in regions with high HCC prevalence, and HBV positive rate in HCC cases in China is as high as 80–90% (17). These data strongly suggest that miR-15b somehow interacts with HBV and may play a role in HBV-related HCC progression. So far, very few studies have been reported on the molecular mechanism of interaction between miR-15b and HBV infection. Therefore, our current study aims to explore the interaction between miR-15b and HBV and to understand the underlying molecular mechanisms.

## MATERIALS AND METHODS

### Cell culture and transfection

Human hepatoma cell lines HepG2, Huh7 and QSG7701 cells were cultured in Dulbecco's modified Eagle's medium containing 10% fetal calf serum, 2 mM L-glutamine, 100 U/ml penicillin and 100 mg/ml streptomycin. HepG2.2.15 cells were cultured in RPMI 1640 containing 380 μg/ml of G418. Cells were maintained in 5% CO_2_ at 37°C. Cells were transfected with plasmids using Lipofectamine^TM^ 2000 (Invitrogen, Carlsbad, CA, USA) following the manufacturer's protocol.

### Quantitative real-time PCR for mRNA/miRNA and for HBV RNA/DNA

Total RNA was extracted with TRIzol reagent (Invitrogen) from cultured cells, liver samples from mice and then reverse transcribed by using Transcript one-step gDNA removal and cDNA synthesis supermix kits (TransGen, Beijing, China) according to instruction. miRNA stem-loop reverse transcription was performed according to a previous study (18). Then, 2 μl of cDNA was used for the quantitative polymerase chain reaction (qPCR) using SYBR Green Realtime PCR Master Mix (TOYOBO, Osaka, Japan) in Eppendorf Mastercycler Realplex Real-time PCR system. U6 snRNA and β-actin were used for normalization of miRNA and mRNA, respectively. Data analysis was performed using the 2^−ΔΔCt^ method (19).

For HBV DNA copy detection, supernatants of HepG2.2.15 or pHBV1.2 (a 1.2-fold full-length HBV genome expression plasmid) transfected Huh-7 cells were harvested, and then HBV DNA was quantified using real-time PCR with HBV PCR assay reagent II (Qiagen, Valencia, CA, USA) following the manufacturer's protocol.

### Construction of plasmid vectors

To construct hepatocyte nuclear factor 1α (HNF1α) expression vector, the full-length HNF1α was amplified from HepG2 cDNA and cloned into pcDNA3.1. An HNF1α silencing vector was constructed by cloning annealed siRNA oligonucleotides targeting HNF1α mRNA into pSilencer-2.1-U6 hygro siRNA expression vector (20). A miR-15b sponges expression plasmid was constructed by inserting a sequence containing six copies of antisense oligonucleotides for mature miR-15b with 4-nt spacers to the pCAGGs vector (21). A miR-15b Gsensor vector for monitoring the function of endogenous miR-15b was constructed by inserting completely complementary sequences of miR-15b into a miRNA target identification reporter plasmid pMIR-luciferase (constructed in this laboratory). Also a reporter plasmid for the 3’UTR of HNF1α was constructed by amplifying the sequence by RT-PCR from RNA of HepG2 and inserting it into the miRNA target identification vector pMIR-luciferase. The rAAV8–1.3HBV (containing 1.3 HBV viral genome) was purchased from Five Plus Molecular Medicine Institute (Beijing, China). Reporter vectors for HBV regulatory fragments were constructed by inserting HBV *cis*-regulation elements (adw2 serotype, accession no. X02763.1; Enhancer I (containing X promoter), nt 2309–2719; Enhancer II (containing C promoter), nt 3034–16; S1p, nt 405–1023; S2p, nt 1154–1342) into pGL3-basic reporter vector (Promega, Madison, WI, USA), respectively. Site-directed mutagenesis of the vectors containing the HBV Enhancer I and 3’UTR of HNF1α gene was performed using the Multipoints Mutagenesis Kit (TaKaRa, Dalian, China) according to the manufacturer's instructions. Primers used in this study were synthesized by Invitrogen (Beijing, China). Sequences for primers and oligonucleotides used in this study are listed in Supplementary Table S1.

### HBV DNA Southern blotting

For intracellular HBV DNA Southern blot detection, HBV DNA replicative intermedi­ates from intracellular core particles were extracted by HBV DNA isolation kit (GenMed Scientifics, USA) according to the instruction and subjected to 1% agarose gel electrophoresis, then transferred onto a positively charged nylon membranes (Millipore, Billerica, MA, USA). The probe specific to the HBx gene sequence prepared by PCR was labeled using a DIG High Prime DNA Labeling and Detection Starter Kit II (Roche, USA), hybridized according to the manufacturer's protocol. The signals were detected on an X-ray film using CSPD (Roche) as a chemiluminescent substrate. HBx gene amplified primers are listed in Supplementary Table S1.

### ELISA assays for HBV surface and e antigen

The extracellular HBV surface antigen and e antigen produced by HepG2.2.15 or Huh-7 transfected with pHBV1.2 cells were detected by the ELISA kit for HBV surface/e antigen made by Kehua (Shanghai, China).

### Western blotting

Cells were collected and treated with RIPA lysis buffer. Lysate in 60 μg protein per lane was separated by 10% sodium dodecyl sulphate-polyacrylamide gel electrophoresis. After electrophoresis, proteins were transferred onto a polyvinylidene difluoride (PVDF) membrane (Millipore, Billerica, MA, USA) and blocked with 5% milk. Then the membrane was incubated with anti-β-actin and HNF1α antibodies (Santa Cruz Biotechnology, Santa Cruz, CA, USA) overnight at 4°C, followed by washing and incubation with a horseradish peroxidase-conjugated secondary antibody (Santa Cruz Biotechnology). The membrane was washed and then detected by enhanced chemiluminescence with Millipore reagents.

### MTT assay

Briefly, cells were trypsinized and seeded into 96-well plates at a density of 9 × 10^3^ cells/well in a volume of 100 μl. The cells were incubated with 20 μl, 5 mg/ml of MTT (Sigma-Aldrich, St. Louis, MO, USA) solution for 4 h under regular culture condition. After the supernatant was removed, 150 μl DMSO was added to dissolve the crystals. The absorbance values at 490 nm were read at specified time points with a BioTek Synergy 2 multiwell spectrophotometer. Viable cells were tested at 0, 1, 2, 3 days after plating, and each experiment was repeated three times.

### Luciferase assay

Luciferase assays were performed using the Dual-Luciferase Reporter Assay System (Promega) 48 h after transfection. Briefly, cells were washed with phosphate buffered saline and lysed chemically. Lysates were then centrifuged to remove cellular debris. Twenty microliter of the supernatants was loaded into a Glomax 20/20 Luminometer (Promega). Then the instrument performed a sequential autoinjection of 100 μl of Luciferase Assay Reagent II (substrate for firefly luciferase) and 100 μl of Stop and Glow Reagent (stop solution for firefly luciferase containing the substrate for Renilla luciferase). The mean of the luciferase activities values measured for 10 s each was used to calculate ratios between Renilla and firefly luciferase.

### Animals and Ethics statement

rAAV8–1.3HBV-transduced mouse model: HBV persistent replication with recombinant Adeno-Associated Virus 8 (AAV, serotype 8) carrying 1.3 copies of HBV genome (rAAV8–1.3HBV) was induced by tail vein injection of rAAV8–1.3HBV (1 × 10^11^ vg/200 μl/mouse) (22,23).

AdGFP-HBx and AdGFP-1.6 HBV-infected mice: 6-week-old C57BL/6 mice were infected by injection with 5 × 10^9^ pfu purified recombinant adenovirus (AdGFP, AdGFP-1.6 HBV and AdGFP-HBx). Mice were sacrificed at day 7 after inoculation, and their livers were snap frozen in liquid nitrogen and stored at −70°C for subsequent analyses.

HBx Transgenic Mouse Model: The liver samples of HBx knocked-in locus of p21 transgenic mice, which developed HCC after the age of 18 months, were kindly provided by Dr Youliang Wang (the Genetic Laboratory of Development and Diseases, Institute of Biotechnology, Beijing, China) (24).

All mice were divided into groups, and each group consisted of five mice. The relative expression of miR-15b of RNA samples and HBV DNA copy detection in mice serum were analyzed by qRT-PCR as described above.

The animal studies were carried out in strict accordance with the recommendations in the guide for the Care and Use of Laboratory Animals of the National Ministry of Health of China. The protocol was approved by the Committee on the Ethics of Animal Experiments of Beijing Institute of Microbiology and Epidemiology IACUCs (Permit No. BIME 2012–96).

### Statistical analysis

Each experiment was repeated at least three times. Quantitative values were expressed as mean ± SEM. Comparison of the relative activity luciferase, mRNA and protein expression levels between each two groups were analyzed with Student's t-test in all cases. All statistical analysis was performed in the GraphPad Prism 5 software, and a *P* value < 0.05 was considered statistically significant.

## RESULTS

### Ectopic miR-15b expression promotes HBV replication and expression

**Figure 1.**
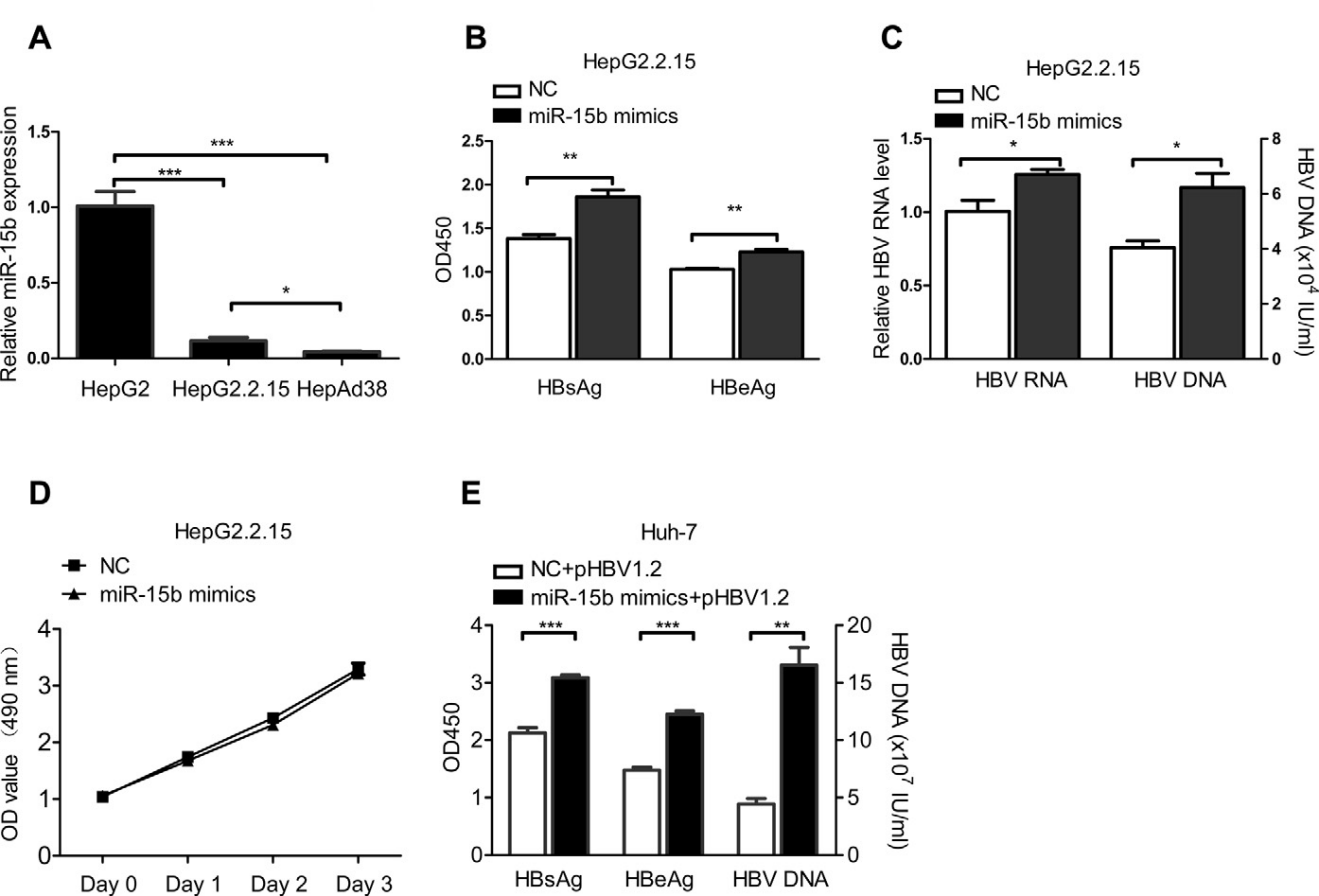
Ectopic miR-15b expression promotes HBV replication and expression. (**A**) MiR-15b levels were detected by qRT-PCR in HepAD38, HepG2.2.15 and HepG2 cells. (**B**) HBsAg/HBeAg, (**C**) HBV RNA/ DNA levels in HepG2.2.15 cells transfected with 40 nM miR-15b mimics or a scrambled NC. (**D**) Proliferation of HepG2.2.15 cells transfected with miR-15b mimics or a scrambled NC. Cell proliferation was evaluated by MTT assay. (**E**) Huh-7 cells were co-transfected with 1.5 μg pHBV1.2 vector and 20 nM miR-15b mimics or a scrambled NC. Culture supernatants and cells were respectively collected for testing HBsAg/HBeAg, supernatants HBV DNA 72 h after transfection. *P < 0.05, **P < 0.01 and ***P < 0.001.

Previously, when we compared the miRNA expression profile in HepG2, HepG2.2.15 and HepAd38 cells, miR-15b was identified as a miRNA whose expression showed an inverse relationship with HBV replication level. This finding was confirmed by real-time PCR (Figure 1A) and indicated that miR-15b may be associated with HBV replication. To test whether miR-15b is involved in the regulation of HBV replication, we overexpressed miR-15b via transfection of miR-15b mimics into HepG2.2.15 cells and then measured HBV RNA, DNA and HBsAg/HBeAg at 72 h after transfection. HBsAg and HBeAg levels (Figure 1B), HBV RNA level and DNA copy number (Figure 1C) were all significantly higher in the miR-15 mimics group than in the scrambled control group. Southern blot and qRT-PCR were used to detect HBV replicative intermediate DNA in HepG2.2.15 cells transfected with miR-15b mimics or a scrambled negative control (NC) (Supplementary Figure S1A). The results indicated that ectopic miR-15b expression increased the HBV replicative intermediate DNA in HBV replication cells.

Next, cell proliferation was evaluated with MTT assays, and the results showed that overexpression of miR-15b did not promote the growth of HepG2.2.15 cells (Figure 1D) and Huh-7 cells used in the next experiment (Supplementary Figure S1B), which is consistent with the reported role of miR-15b as a cell cycle checker (25). Taken together, these data suggest that miR-15b promotes HBV replication and expression through intracellular interaction, not through promoting cell proliferation.

We then performed similar experiments in Huh-7 cells with transient transfection of pHBV1.2 plus miR-15b mimics or the scrambled control. The results for HBsAg/HBeAg level, HBV DNA copy number (Figure 1E) and HBV replicative intermediate DNA (Supplementary Figure S1C) were consistent with the observations in HepG2.2.15 cells.

### Suppression of endogenous miR-15b inhibits HBV replication and expression

**Figure 2.**
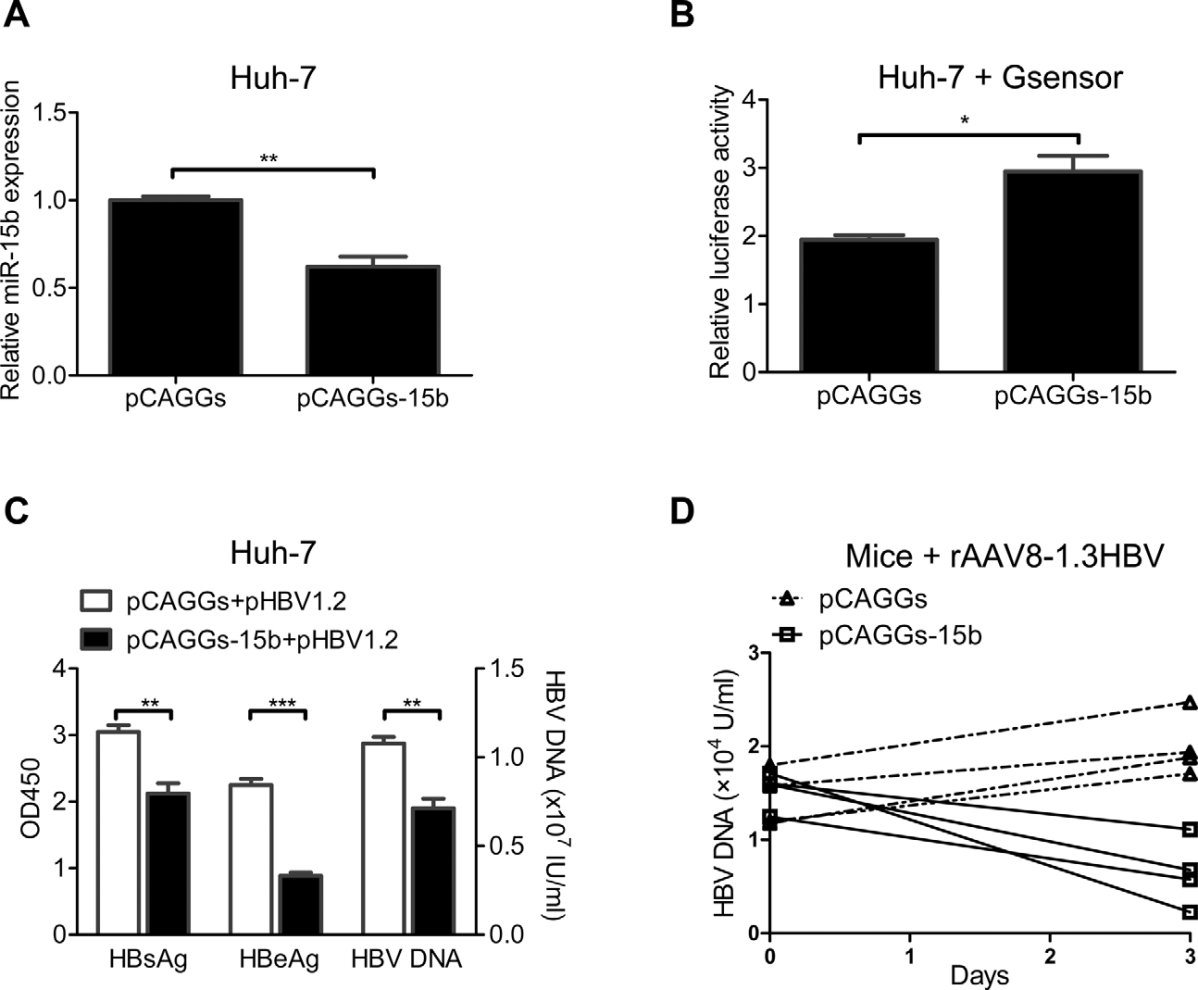
Suppression of endogenous miR-15b inhibits HBV replication and expression. (**A**) Huh-7 cells were transfected with 3 μg pCAGGs-15b or the control vector pCAGGs, and endogenous miR-15b level was detected 72 h later. (**B**) Huh-7 cells were co-transfected with 0.1 μg pMIR-15b Gsensor, 0.1 μg pRL-TK and 1 μg pCAGGs-15b or pCAGGs. Dual-luciferase reporter assays were performed. (**C**) Huh-7 cells were co-transfected with 1.5 μg pCAGGs-15b and 1.5 μg pHBV1.2, and culture supernatants were assayed for HBsAg/HBeAg and HBV DNA 72 h later. (**D**) HBV-expressing mice (infected with rAAV8–1.3HBV) were given pCAGGs-15b sponges or pCAGGs via hydrodynamic injection, and HBV DNA was detected 3 days later. *P < 0.05, **P < 0.01 and ***P < 0.001.

We then studied the effect of decreased miR-15b level on HBV replication and expression. We first validated that transfection of pCAGGs-15b sponges (‘absorbs’ miR-15b) into Huh-7 cells could lower intracellular concentration of free miR-15b as compared to pCAGGs (Figure 2A). The effect of pCAGGs-15b sponges on miR-15b function was also confirmed with a miR-15b Gsensor luciferase reporter plasmid. Luciferase assay showed that pCAGGs-15b sponges could effectively inhibit the function of endogenous miR-15b (Figure 2B).

The validated miR-15b knockdown tool, pCAGGs-15b sponges, was then co-transfected into HepG2.2.15 cells and Huh-7 cells with pHBV1.2 to observe the effect of miR-15b down-regulation on HBV replication and expression. As shown in Figure 2C, the pCAGGs-15b sponges group had significantly lower HBeAg/HBsAg expression and HBV DNA replication than the control pCAGGs group. In HepG2.2.15 cells, however, no differences were observed between the two groups for these parameters (data not shown), which might be explained by the already very low level of miR-15b in HepG2.2.15 cells (see Figure 1A).

In addition, the effect of miR-15b down-regulation on HBV was observed in a persistent HBV expression mouse model: rAAV8–1.3HBV infected-mouse (22,23). We first evaluated the efficiency of pCAGGs-15b transduction after hydrodynamic injection and found that pCAGGs-15b was able to effectively inhibit *in vivo* expression of endogenous miR-15b, while it promoted *in vivo* expression of HNF1α, the target of miR-15b (Supplementary Figure S2A and S2B). Further, we found that all mice in the pCAGGs-15b group showed decreased levels of HBV DNA as compared to their day 0 levels; in contrast, mice in the pCAGGs group had levels comparable to, or slightly higher than, their day 0 levels (Figure 2D), indicating that miR-15b down-regulation inhibits HBV replication and expression *in vivo*.

### MiR-15b promotes HBV Enhancer I activity through HNF1α

We sought to identify the relevant endogenous targets of miR-15b that might affect HBV expression and replication. We first reasoned that miR-15b does not directly interact with HBV transcripts, as such interaction would most likely inhibit HBV transcription. Nevertheless, we tested with cell culture experiments for a putative miR-15b binding sequence within the HBV genome, as predicted by bioinformatics, and confirmed its non-interaction with miR-15b (data not shown).

**Figure 3.**
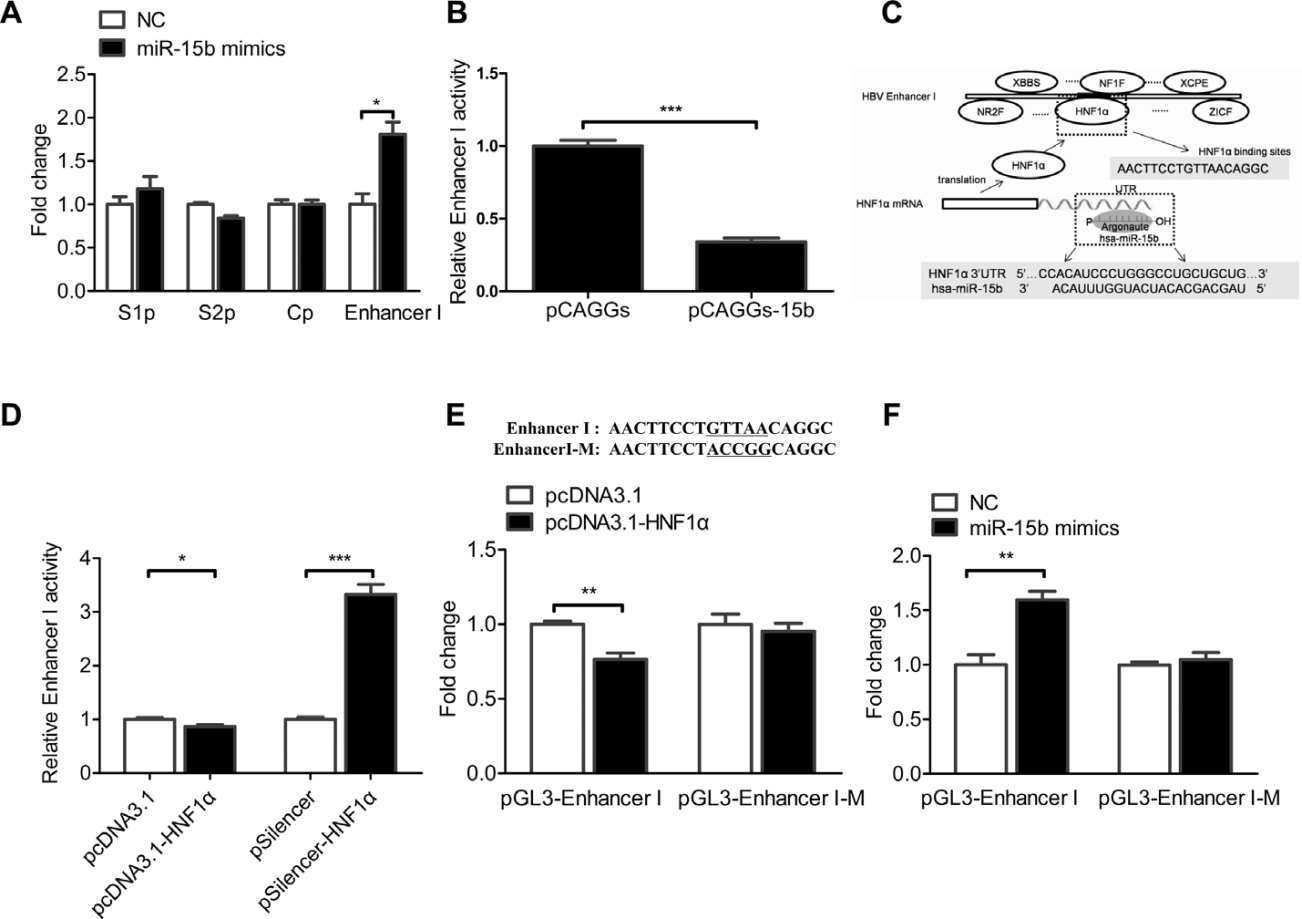
MiR-15b promotes HBV Enhancer I activity through HNF1α. All experiments were performed with dual-luciferase reporter assay in Huh-7 cells 48 h after transfection. (**A**) The reporter luciferase was driven by a HBV promoter (S1p, S2p, or Cp) or Enhancer I. Cells were co-transfected with 10 nM miR-15b mimics or control. (**B**) Cells were co-transfected with the HBV Enhancer I-driven luciferase reporter plasmid and another vector (pCAGGs-15b) related to miR-15b activity. (**C**) HNF1α is the putative miR-15b target, as predicted by Genomatix and Targetscan with two restrictions: (i) transcription factors of Enhancer I and (ii) 3’-UTR targeted by miR-15b. (**D**) Cells were co-transfected with the HBV Enhancer I-driven luciferase reporter plasmid and another vector (pcDNA3.1-HNF1α or pSilencer-HNF1α). (**E** and **F**) The luciferase reporter was driven by the wild-type or the mutant HBV Enhancer I. The co-transfected vector was related to HNF1α (E) or miR-15b (F). *P < 0.05, **P < 0.01 and ***P < 0.001.

Then, we tested the interaction of miR-15b with regulatory regions of the HBV genome (26,27).The promoter and enhancer sequences of HBV genes were respectively cloned into a luciferase reporter plasmid pGL3-basic. These plasmids were respectively co-transfected with a normalizing luciferase plasmid pRL-TK and miR-15b mimics, or a control miRNA, into Huh-7 cells. Reporter activity of the pGL3-Enhancer I was significantly higher in the miR-15b mimics group than in the control miRNA group, while no differences were found in other reporter vectors (Figure 3A), indicating miR-15b indirectly promotes the activity of HBV Enhancer I.

The activity of Enhancer I was then tested in Huh-7 cells using pCAGGs-15b sponges. As expected, the activity of Enhancer I-driven luciferase reporter was lower in the pCAGGs-15b sponges group than in the control group, confirming that miR-15b positively correlates with Enhancer I activity (Figure 3B).

After identifying the end target of miR-15b as HBV Enhancer I, the direct target(s) of miR-15b was then predicted by bioinformatics with two restrictions: (i) transcription factors of Enhancer I and (ii) 3’-UTR targeted by miR-15b. Prediction returned HNF1α as the only promising target (Figure 3C).

The relationship of HNF1α and HBV Enhancer I was first confirmed in Huh-7 cells by testing the effect of up- or down-regulation of HNF1α on Enhancer I activity using the Enhancer I reporter plasmid. Overexpression of HNF1α resulted in lower Enhancer I activity, while knocking down HNF1α level with a silencer dramatically increased Enhancer I activity (Figure 3D), confirming that HNF1α is a negative regulator of HBV Enhancer I.

We compared the activity of wild-type or mutant Enhancer I (changed from GTTAA to ACCGG in the HNF1α binding site) in Huh-7 cells and found that the activity of mutant Enhancer I was significantly higher than that of the wild-type Enhancer I (Supplementary Figure S3A). It is possibly because the endogenous HNF1α inhibits Enhancer I activity, but it does not affect the activity of Enhancer I when HNF1α's binding site is mutated. Then, the effect of HNF1α up- or down-regulation on wild-type or mutant Enhancer I was tested. The results showed that mutant Enhancer I abolished the inhibitory effect of HNF1α on Enhancer I activity (Figure 3E and Supplementary Figure S3B), confirming the direct interaction between HNF1α and HBV Enhancer I.

Next, the effect of miR-15b was tested with the HNF1α-binding-site-mutant Enhancer I reporter plasmid (Figure 3F). With the wild-type Enhancer I reporter plasmid, miR-15b mimics resulted in increased activity similar to that shown in Figure 3A. With the HNF1α-binding-site-mutant reporter plasmid, however, the luciferase activity did not differ between miR-15b and the scrambled miRNA (Figure 3F). These data suggest that the effect of miR-15b on Enhancer I requires HNF1α interaction with Enhancer I.

### MiR-15b directly inhibits the expression of HNF1α by binding to its 3’-UTR

**Figure 4.**
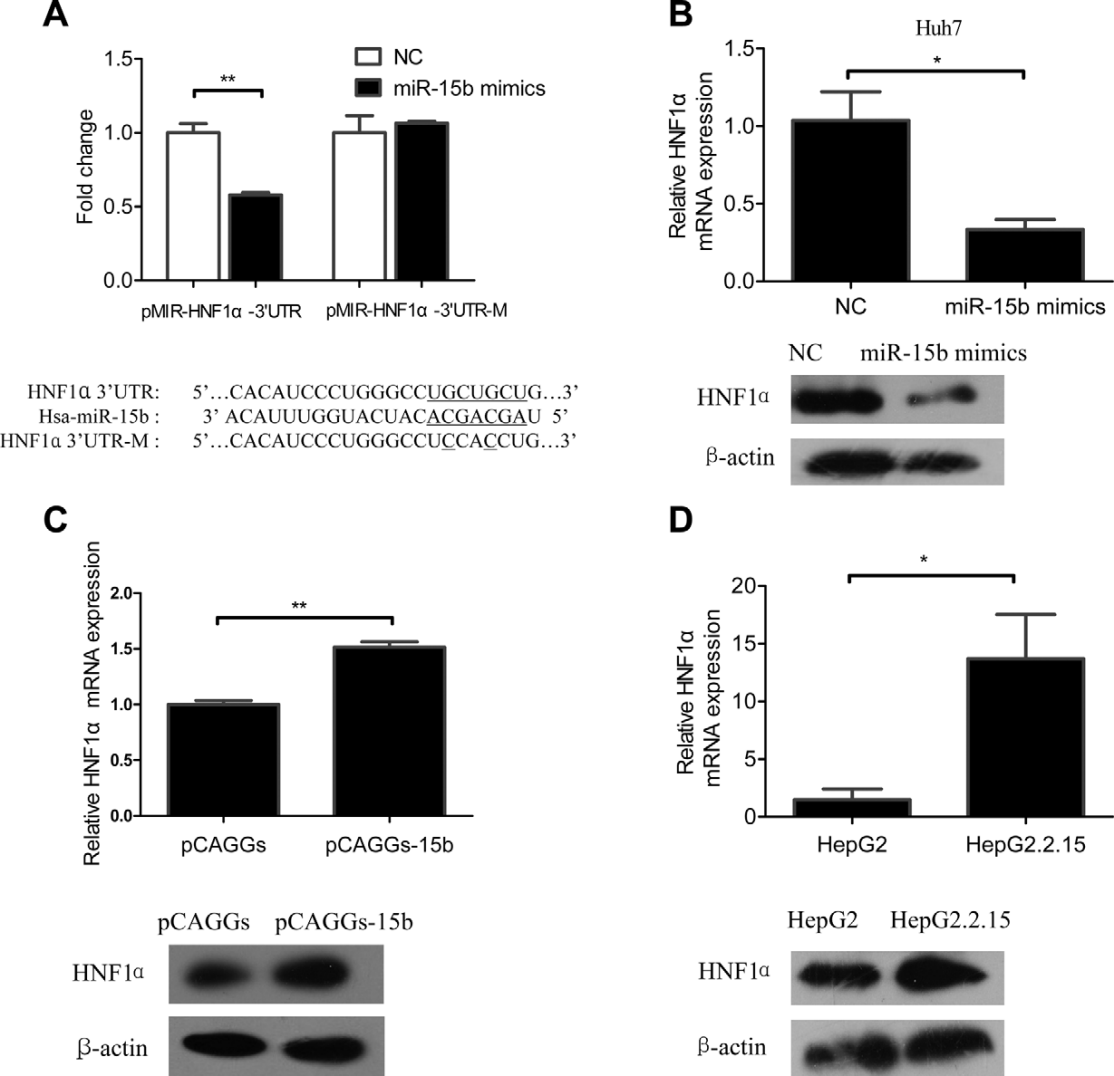
MiR-15b directly inhibits the expression of HNF1α by binding to its 3’-UTR. (**A**) Upper panel: Dual-luciferase reporter assay of Huh-7 cells transfected as indicated. Lower panel: Alignment of miR-15b and HNF1α 3’-UTR sequence, showing the wild-type and a mutant form of miR-15b target sequence. (**B**) Endogenous expression of HNF1α mRNA (upper panel) and HNF1α protein (lower panel) in Huh-7 cells transfected with 40 nM miR-15b mimics/NC. (**C**) Endogenous expression of HNF1α mRNA (upper panel) and HNF1α protein (lower panel) in Huh-7 cells transfected with 3 μg pCAGGs-15b. (**D**) Endogenous expression of HNF1α mRNA (upper panel) and HNF1α protein (lower panel) in HepG2 and HepG2.2.15 cells with no transfections. β-actin was used as internal controls. *P < 0.05 and **P < 0.01.

To test whether miR-15b interacts directly with HNF1α through binding to its 3’-UTR, as predicted by bioinformatics, the wild-type and mutant HNF1α 3’-UTR, with 3 nt mutations within the 7 nt base-pairing site, were prepared (Figure 4A). Two reporter plasmids were constructed carrying either the wild-type or the mutant 3’-UTR of HNF1α downstream of the luciferase gene. Then the dual-luciferase reporter assay was performed in Huh-7 cells with the addition of miR-15b mimics or a control miRNA. As shown in Figure 4A, addition of miR-15b mimics resulted in a significant decrease in activity for the reporter carrying the wild-type HNF1α 3’-UTR, but did not affect the reporter activity with the mutant HNF1α 3’-UTR. This result indicates that HNF1α is a direct target of miR-15b and that the 7 nt base-pairing sites in its 3’-UTR were where miR-15b binds HNF1α transcripts.

To further determine the effect of miR-15b on HNF1α, a series of experiments were performed to examine the relationship between cellular miR-15b level and endogenous HNF1α mRNA and protein expression. First, in Huh-7 cells, the endogenous HNF1α mRNA and protein expression levels at 72 h after miRNA mimics transfection were significantly lower in the miR-15b group than the scrambled control group (Figure 4B). A similar phenomenon was also observed in HepG2.2.15 cells (Supplementary Figure S4). Inversely, knockdown of endogenous miR-15b by pCAGGs-15b resulted in the elevated expression of HNF1α (Figure 4C) in Huh-7 cells. Further, the endogenous HNF1α mRNA and protein expression levels in HepG2 cells were dramatically lower than those in HepG2.2.15 cells (Figure 4D), which correlates inversely with endogenous miR-15b level in these two cells (Figure 1A). These results indicated that miR-15b could directly reduce HNF1α expression through degradation of its mRNA transcripts.

### HNF1α mediates the promotion of HBV Enhancer I activity by miR-15b

**Figure 5.**
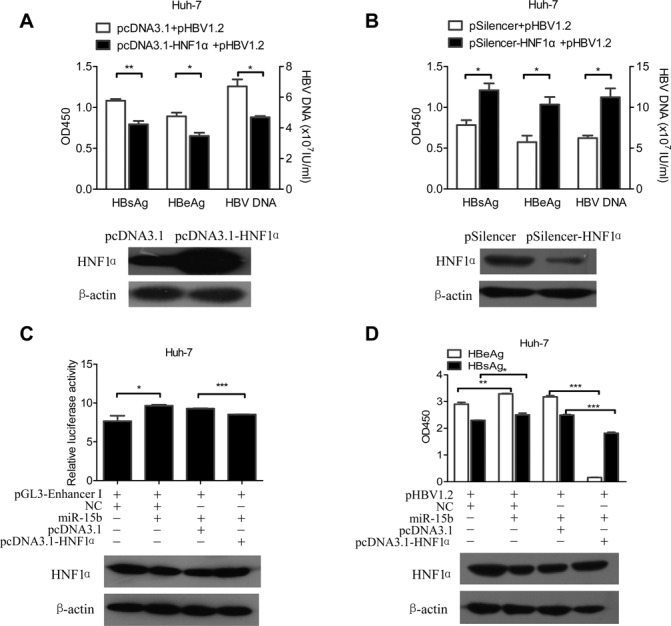
HNF1α mediates the promotion of HBV replication and expression by miR-15b. Huh-7 cells were co-transfected with 1.5 μg pHBV1.2 and 1.5 μg pcDNA3.1-HNF1α /pcDNA3.1 (**A**) or pSilencer-HNF1α/pSilencer (**B**). HBsAg/HBeAg/HBV DNA (upper panel) and HNF1α protein (lower panel) were detected. Huh-7 cells were transfected with expressing vectors as indicated. The relative Enhancer I activity (**C**, upper panel), HBsAg/HBeAg (**D**, upper panel) and HNF1α protein (C and D, lower panels) were detected at 48 h post-transfection. *P < 0.05, **P < 0.01 and ***P < 0.001.

Data in Figures 3 and 4 have already indicated HNF1α as the link between miR-15b and HBV Enhancer I activity. Here we carried out further experiments to solidify this link. First, we tested whether changing HNF1α levels would have the opposite effect on HBV replication and expression as changing miR-15b. HNF1α level in Huh-7 cells was either increased by overexpression (Figure 5A, lower) or decreased by gene silencing (Figure 5B, lower), and we found that HBV expression and DNA replication were decreased if HNF1α was increased (Figure 5A, upper), but increased if HNF1α was decreased through silencing (Figure 5B, upper). The result shown in Figure 5A is similar to that in Figure 2C, where miR-15b was knocked down, and the result shown in Figure 5B is similar to that in Figure 1B and E, where miR-15b was overexpressed. Taken together, these data, when considering that HNF1α is the direct target of miR-15b, as shown in Figure 4, suggest that HNF1α mediates the promotion of HBV activity by miR-15b.

We next tested the interplay among miR-15b, HNF1α and HBV Enhancer I in one experiment. In a dual-luciferase reporter assay system, where the reporter gene carries the Enhancer I sequence, increasing miR-15b level (by miR-15b mimics) in the system increased reporter activity, but further addition of HNF1α (Figure 5C, lower) (by transfection of an expression plasmid) brought the reporter activity down, almost abolishing the increase brought by miR-15b (Figure 5C, upper). Similarly, in pHBV1.2 transfected Huh-7 cells, addition miR-15b mimics to the cells increased both HBeAg and HBsAg expression, while overexpression of HNF1α (Figure 5D, lower) brought these antigen expression levels down, with a dramatic reduction in HBeAg and a significant reduction in HBsAg, as compared with the miR-15b mimics group (Figure 5D, upper).

### The reciprocal regulation between miR-15b and HBV may help to control the level of HBV replication and play a role in persistent HBV infection

**Figure 6.**
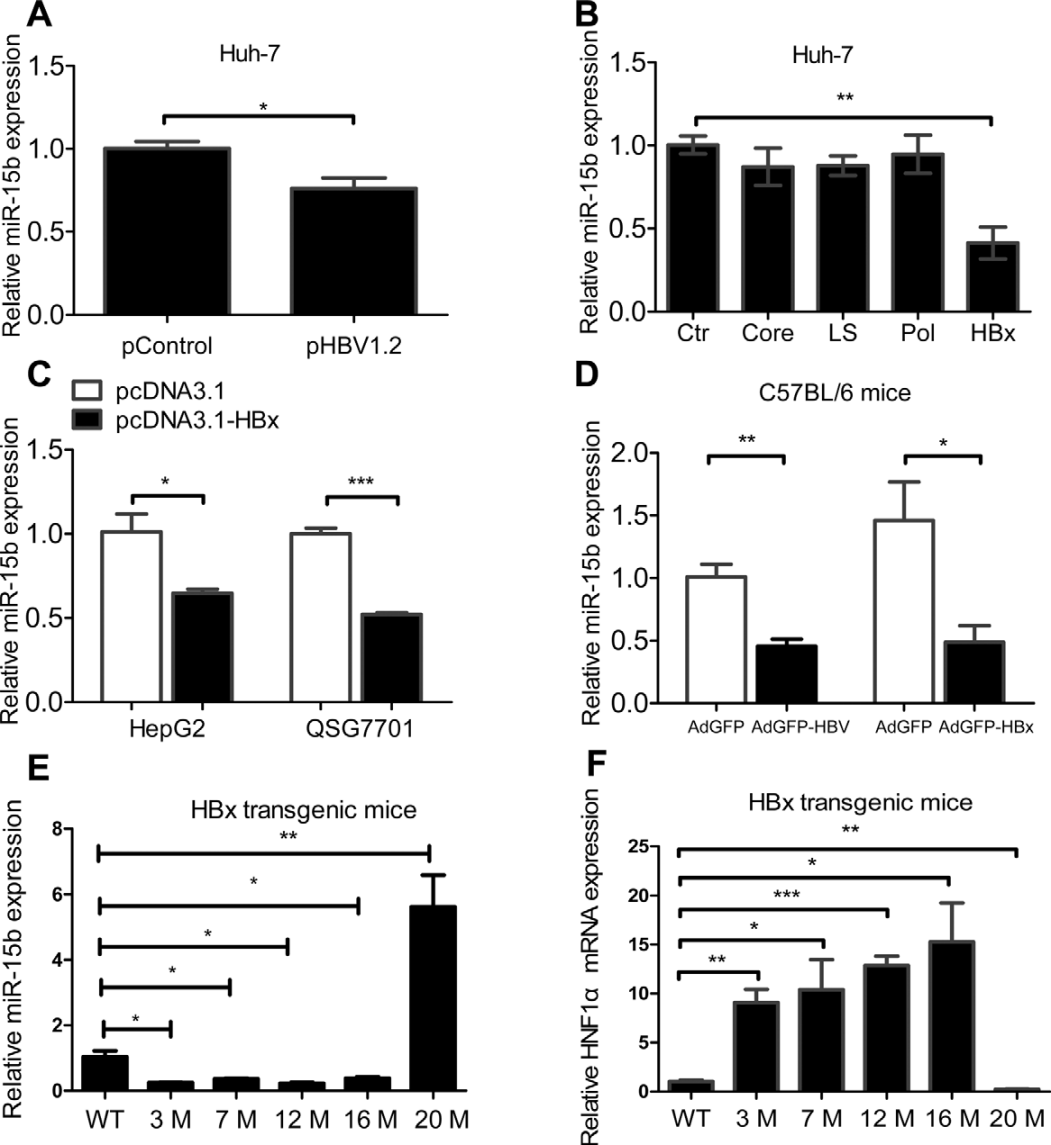
Expression of miR-15b is down-regulated in HBV/HBx-producing cells or mice. (**A**) Huh-7 cells transfected with 3 μg pHBV1.2 or control vector at 72 h post- transfection; (**B**) Huh-7 cells transfected with 3 μg expression vectors for HBV proteins, including Core, LS, Pol, HBx and the negative control (Ctr); (**C**) HepG2 and QSG7701 cells transiently transfected with 3 μg pcDNA3.1-HBx or control; and (**D**) mice at 7 days after receiving hydrodynamic injection of Ad-HBV or Ad-HBx. Assays were performed in triplicate, and miRNA level was normalized to that of U6 snRNA. Relative miR-15b expression (**E**) and HNF1α expression (**F**) was detected in the livers of p21-HBx transgenic mice at different ages (3, 7, 12, 16 and 20 months). WT: Wild-type mice. *P < 0.05, **P < 0.01 and ***P < 0.001.

Our experiments, as described above, showed that miR-15b was lowly expressed in HBV expression cells (Figure 1A). To test whether HBV regulates miR-15b expression, we further detected the expression of miR-15b in Huh-7 cells 72 h after transient transfection with pHBV1.2, or the control vector. Similar to stable HBV-producing HepG2.2.15 cells, Huh-7 cells with transient expression of HBV also had a level of miR-15b expression lower than that of the control cells (Figure 6A). These data confirmed that miR-15b is down-regulated in HBV-producing cells.

To determine which protein encoded by four HBV genes (Pol, LS, X and Core) correlates with miR-15b repression, we examined the expression of miR-15b in Huh-7 cells transiently expressing individual HBV genes, and found that miR-15b expression was significantly lower in cells transfected with HBx, but not with other HBV genes (Figure 6B), indicating that HBx might cause the down-regulation of miR-15b expression. To confirm the effect of HBx on miR-15b expression level, we detected miR-15b in HepG2 and QSG7701 cells transiently transfected with pcDNA3.1-HBx, and miR-15b expression was, indeed, found to be significantly lower in the HBx-expressing cells compared to the control cells for both cell lines (Figure 6C). We further compared miR-15b expression in liver tissue of C57BL/6 mice infected with AdGFP-1.6 HBV/AdGFP-HBx or AdGFP vector, and miR-15b expression in both AdGFP-1.6 HBV and AdGFP-HBx groups was significantly lower than that in the AdGFP group (Figure 6D).

To further study the kinetic expression of miR-15b in HBx transgenic mice from early stage to LC/HCC stage, we detected the miR-15b expression in the livers of HBx-transgenic mice at different ages (24). Compared with wild-type mice, results showed that miR-15b expression decreased at 3, 7, 12 and 16 months, but was elevated at 20 months (Figure 6E), while its target HNF1α expression increased at 3, 7, 12 and 16 months, but was decreased at 20 months (Figure 6F). The decreased miR-15b level in the early stages of HBV/HBx-expressing mice is consistent with our results showing that HBV/HBx down-regulates miR-15b in cells. The HBx transgenic mice develop HCC after 18 months (24). Interestingly, the expression of miR-15b is elevated in the later stages in the mice model. The results are consistent with previously reported clinical observations that miR-15b is elevated at LC or HCC stage (14,16). These experiments showed that the regulation of miR-15b expression may differ at different stages of HBV infection and pathogenesis. HBV direct down-regulates miR-15b in the early stages of infection. Another unknown mechanism may be involved in the up-regulation of the expression of miR-15b during later and pathological stages of HBV infection, such as LC and HCC.

**Figure 7.**
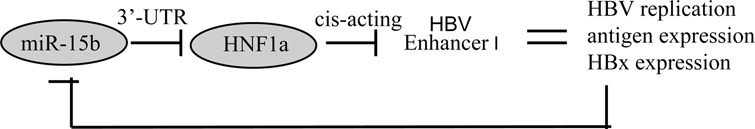
Schematic illustration of the reciprocal regulation between miR-15b and HBV. HNF1α serves as a negative regulator of HBV Enhancer I. Overexpressed miR-15b can suppress the activity of HNF1α, resulting in the up-regulation activity of HBV Enhancer I, which promotes HBV replication and expression of HBV antigen. At the early stage of HBV infection, the increased HBV replication and expression of HBV antigens, particularly the HBx protein, is able to down-regulate miR-15b expression.

Overall, the results shown support the following cascade of events, miR-15 directly binds HNF1α mRNA to attenuate HNF1α expression and thus the inhibitory effect of HNF1α, resulting in transactivation of HBV Enhancer I, in turn causing the enhancement of HBV replication and expression of HBV antigens, including HBx protein, finally leading to the down-regulated expression of miR-15b. A summary of the interplay among HBV, miR-15b and HNF1α is presented in Figure 7.

## DISCUSSION

In this study, we demonstrated that miR-15b promotes HBV replication by targeting HNF1α; a negative regulator of HBV Enhancer I and miR-15b expression in hepatocytes is down-regulated by HBV, and more specifically, by HBx, at the early stages of HBV infection. Our data provide mechanistic detail and insights that support a coherent theory of miR-15b function in HBV-infected hepatocytes and liver tissues.

MiR-15b has been found to be highly expressed in HCC (14) and several other tumors, such as malignant melanoma (28), colonic carcinoma (29) and cervical carcinoma (30). Elevated serum level of miR-15b has been suggested as a biomarker for HCC (16). These association studies pointed out the potential importance of miR-15b in cancers. Here, we focused our study on the role of miR-15b in HCC tissue and hepatocytes, as miR-15b has been found in multiple studies to be a miRNA with the greatest fold change in expression level, thus showing potential clinical value for HCC. In fact, it was reported to have a predictive value for HCC greater than 90% in Asian HBV-infected patients (16).

While miR-15b was reported to be elevated in HCC tissues or serum of HCC patients, miR-15b has been found to be down-regulated in some cell lines with HBV infection (13). In the current study, we observed down-regulation of miR-15b in HBV-infected or HBx-expressing hepatocytes, including HepG2.2.15, Huh-7, HepG2 and QSG7701 cells, and in mice liver tissue expressing HBV or HBx. Results from kinetic expression of miR-15b in HBx transgenic mice have shown that miR-15b level is decreased in the early stages and elevated in the later stages of HBx expression which develop HCC. These observations appear to reconcile with the view that miR-15b is down-regulated upon acute HBV infection and is elevated during later and pathological stages of HBV infection, such as LC and HCC. A dozen or so miRNAs with a function in HBV infection previously showed such gradient expression pattern (31), either elevated or down-regulated at the later stage, and some have been suggested to be potential markers of HBV related liver diseases. Clearly, miR-15b can be added to this list.

The miR-15b belongs to the miR-15/16 family of miRNAs. The miR-15/16 family is composed of miR-15a, miR-15b and miR-16. The miR-15a/16–1 and miR-15b/16–2 gene clusters are located on human chromosomes 13q and 3 (32). These two clusters are highly conserved among mammalian species (33) and have been shown to play very important roles in regulating cell proliferation and apoptosis by targeting cell cycle proteins and the anti-apoptotic Bcl-2 gene (34–36). Specifically, miR-15b has been found to be a direct transcriptional target of E2F, which, in turn, targets cyclin E to reduce its expression and inhibit G1/S transition (25). In addition, miR-15b has also been shown to augment TRAIL-induced HCC cell apoptosis via down-regulating Bcl-w protein expression (14). These cell cycle-arresting and apoptosis-promoting roles suggest that miR-15b has tumor suppressor function which might help to explain why miR-15b overexpression in HCC tissues is related to lower risk of HCC recurrence following curative resection.

HNF1α is an atypical homeodomain-containing transcription factor that transactivates liver-specific genes, including albumin, α-1-antitrypsin, as well as α-and β-fibrinogen. It has been documented that HNF1α transactivates HBV Enhancer II/core promoter and large surface antigen promoter (37,38). In the present study, we evaluated the impact of HNF1α on all HBV promoters and Enhancers and also showed that HNF1α could promote the activity of core promoter and S1 promoter (data was not shown), consistent with the above reports (37,38). Notably, we found that HNF1α decreased the activity of HBV Enhancer I (Figure 3), while HBV Enhancer I is one of the key regulation regions that controls the level of HBV replication (27). It has been reported that mutations in the Enhancer I region of HBV have a major impact on HBV replication (39) and Enhancer I greatly increases the activity of S1, S2 and core promoters (40,41). We thus believe that although HNF1α elevates the activity of Enhancer II/core promoter or S1 promoter, the major effects of HNF1α influence on the level of HBV replication may depend on its influence on the activity of Enhancer I.

It has been reported that HBx protein can stimulate the DNA binding activity of HNF1α (42). The current study confirmed this observation and arrived at the finding that miR-15b binds to HNF1α 3’-UTR, resulting in the degradation of HNF1α transcripts. This role of miR-15b in the interaction between HBx and HNF1α appears to be a fine tuning in that HBx not only binds directly to HNF1α to enhance its activity but also decreases its negative regulator miR-15b to increase HNF1α expression. The effects of HBx on both miR-15b and HNF1α point to the same purpose: lowering or stabilizing HBV replication and expression. However, we could not rule out the possibility that miR-15b represses the expression level of HNF1α by targeting an unknown enhancing factor of HNF1α. Besides miR-15b, all other nine mature miRNAs in miR-15/107 seed family (43) may also affect HNF1α because they all have the same seed sequence.

As previously noted (31), three miRNAs (miR-210, miR-199a-3p and miR-125a-5p) have been reported to interact directly with HBV transcripts, while the other miRNAs indirectly regulate HBV life cycles by influencing relevant cellular proteins. For miRNAs promoting HBV replication, it appears that they share a common pattern of targeting a negative cellular regulator of HBV activity. For example, miR-501 targets HBXIP (44), and miR-372/373 targets NFI/B (45). MiR-15b acts in a similar fashion. An exception is miR-1 which enhances a positive regulator, FXRA (46). For miRNAs which suppress HBV replication, such as miR-210, miR-199a-3p (47), miR-125a-5p (48), miR-29c (49), miR-122 (50,51) and miR-141 (52), besides the three which directly target HBV transcripts, most act through targeting a positive regulator of HBV, such as miR-141, which targets peroxisome proliferator-activated receptor alpha (PPARA).

Some groups have explored the mechanisms how HBV regulates miRNAs. For miR-122 (53) and miR-15a (54,55), HBV mRNA harboring complementary sites act as sponges to bind and sequester endogenous miRNA, indicating that the highly redundant HBV transcripts are involved in HBV-mediated miRNA suppression. G. Wu *et al.* reported that c-Myc mediated the HBx-induced repression of miR-15a/16 in HepG2 cells (32), while C.S. Wu *et al.* (56) presumed that regulation of miR-15b by HBx may also be mediated by HBx RNA as the mode of HBV regulation on miR-15a because miR-15a and miR-15b share most of the same sequence. In consistent with the above reports (32,56), our results have demonstrated that HBx, rather than other HBV components, suppresses miR-15b expression in hepatocytes. Both miR-15b and miR-16–2, which are in the same cluster and have a common promoter, are down-regulated by HBx. Therefore, an unknown transcription repressor may be involved in the mechanism how HBx down-regulates miR-15b, which needs further investigation.

The current study enhances our understanding of the physiology and pathogenesis of HBV infection. The mouse infection models employed in the current study provide good models of the early stage, or carrier state, of HBV infection. At this stage, HBV/HBx down-regulates miR-15b, a promoter of HBV replication and expression. The overall effect of this feedback loop is to maintain a moderate level of HBV activity. While the expression of miR-15b is elevated in LC and HCC, we know HBV is not highly expressed at those stages. This suggests that the regulatory loop found in the current study has somehow been broken at the later, pathogenic stage and that miR-15b no longer promotes HBV replication. The dual role of miR-15b, i.e. promoting HBV replication at early stage, but anti-proliferation and proapoptosis at later stage, of HBV infection, suggests that miR-15b is likely to be a good indicator of HBV-related disease progression. This could explain why miR-15b and miR-130 (down-regulated in HCC tissues in contrast to miR-15b) could be used as a set of highly accurate markers for HBV-related HCC (16). In addition, miR-15b could be used as an investigational tool in cells to manipulate HBV activity and sensitize cells to apoptosis in HCC.

In hepatocytes, we have provided both *in vitro* and *in vivo* evidence that miR-15b promotes HBV Enhancer I activity by targeting HNF1α, a direct binding negative regulator of HBV Enhancer I, and that HBx down-regulates the level of intracellular miR-15b. Consequently, this work adds to the body of knowledge concerning complex interactions between HBV and host miRNAs. The identification of HBV-modulating miRNAs and their target genes has potential clinical and investigational implications.

## SUPPLEMENTARY DATA

Supplementary Data are available at NAR Online.

## FUNDING

National Program of Infectious Diseases [2012ZX10004–502]; National Science Foundation of China [30900753]. Funding for open access charge: National Program of Infectious Diseases.

*Conflict of interest statement*. None declared.

## Supplementary Material

SUPPLEMENTARY DATA
