# Supplementary material for: Modulation of HBV replication by microRNA-15b through targeting hepatocyte nuclear factor 1α
Source: Nucleic Acids Res. 2014 Apr 4;42(10):6578–90. doi: 10.1093/nar/gku260 (PMC4041434; doi:10.1093/nar/gku260)
Supplement: SUPPLEMENTARY DATA [file supp_42_10_6578__index.html]

SUPPLEMENTARY DATA 

# Modulation of HBV replication by microRNA-15b through targeting hepatocyte nuclear factor 1α

## SUPPLEMENTARY DATA

**Files in this Data Supplement:**

- SUPPLEMENTARY DATA
