# Supplementary material for: Modulation of HBV replication by microRNA-15b through targeting hepatocyte nuclear factor 1α
Source: Nucleic Acids Res. 2014 Apr 4;42(10):6578–90. doi: 10.1093/nar/gku260 (PMC4041434; doi:10.1093/nar/gku260)
Supplement: SUPPLEMENTARY DATA [file supp_gku260_nar-03375-v-2013-File009.pdf]

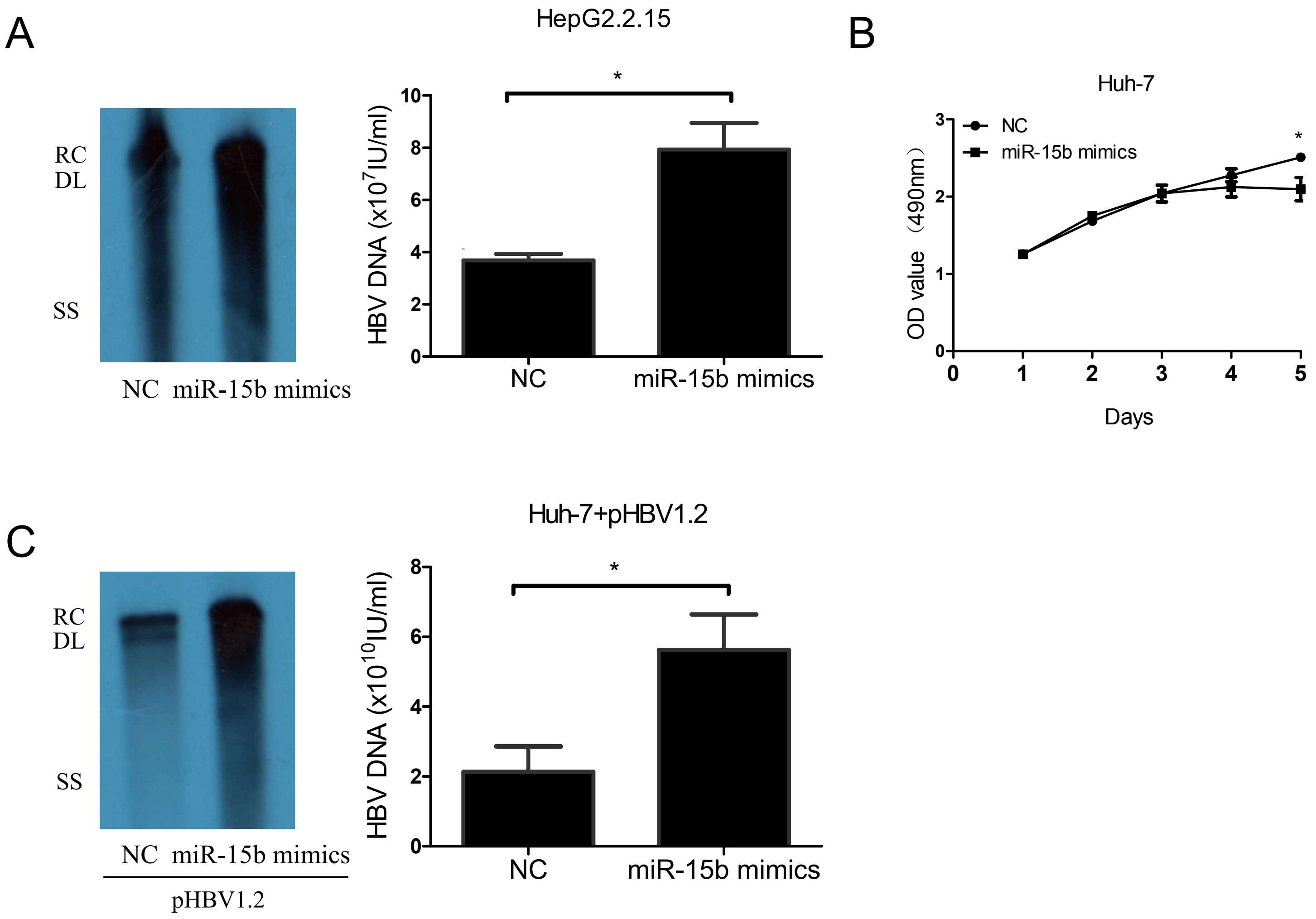

Supplementary Fig. 1 (Fig. S1)

A

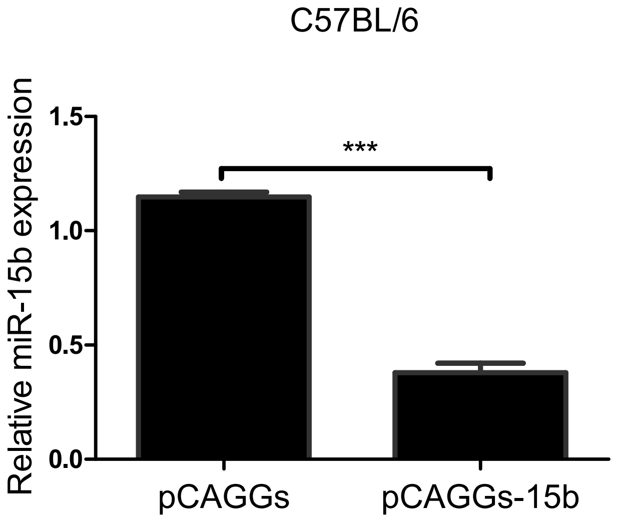

B

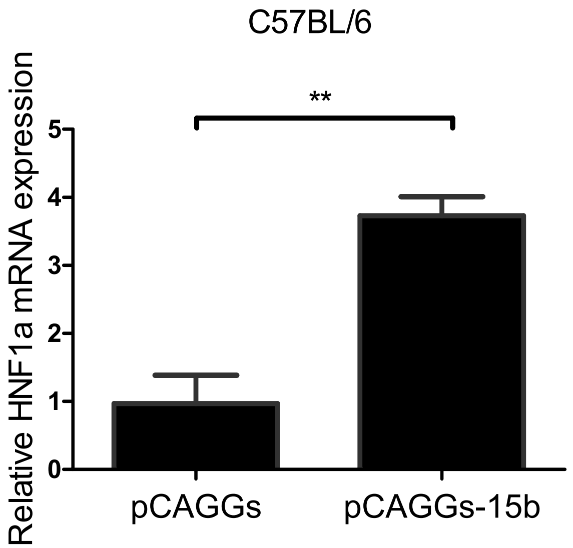

Supplementary Fig. 2 (Fig. S2)

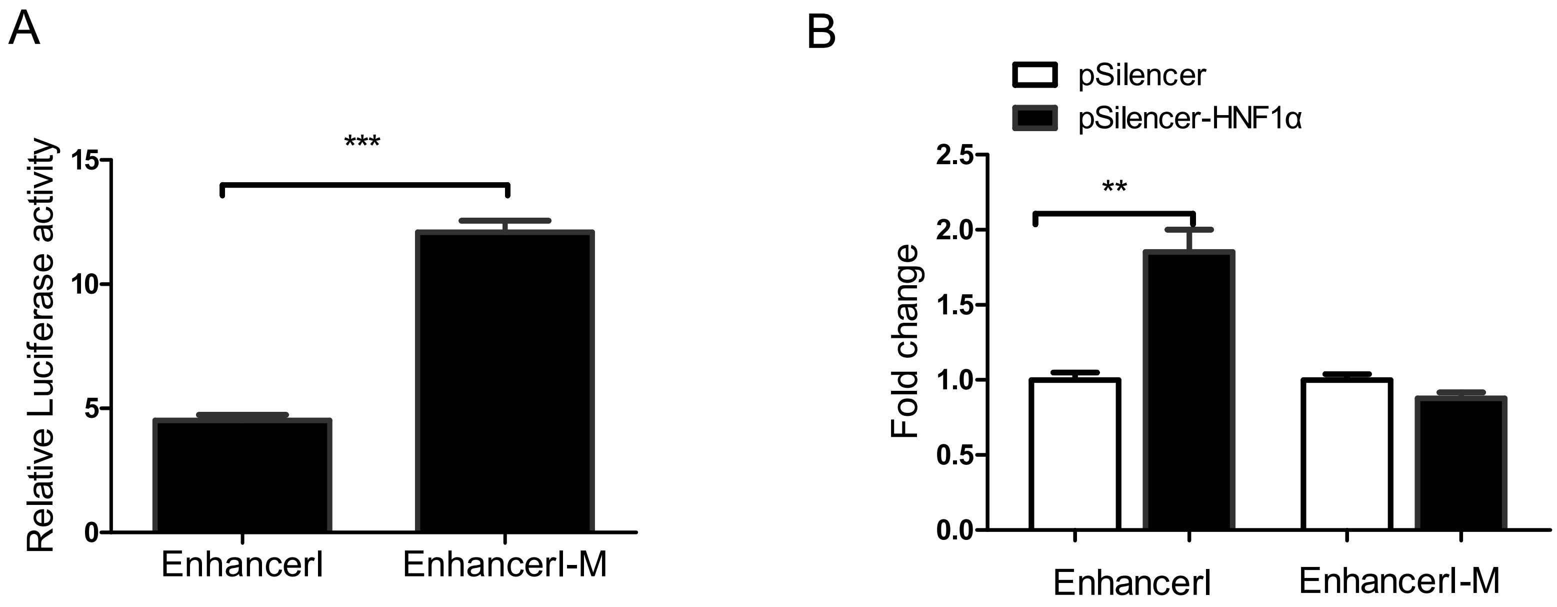

Supplementary Fig. 3 (Fig. S3)

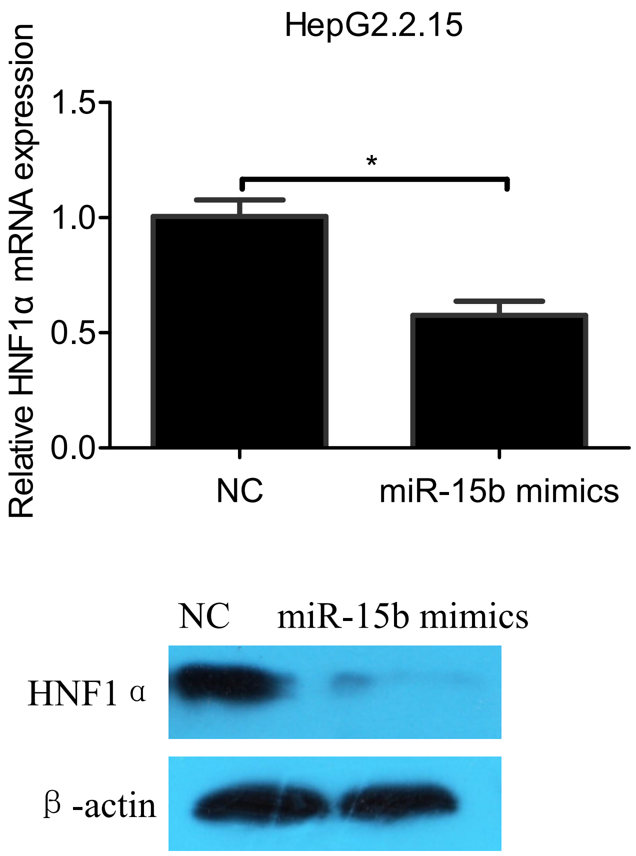

Supplementary Fig. 4 (Fig. S4)

Table S1 Oligonucleotide sequences for miRNAs/siRNAs and primers used for real-time PCR and vector cloning.

Gene name	Application	Type	Sequence 5’-3’
U6	Reverse transcription	stem-loop RT	GTCGTATCCAGTGCAGGGTCCGAGGTATTCGCACTGGTACGACAAAAATATG
	real time PCR	forward	CGCAAATTCGTGAAGCGTTC
		Universal reverse	GTGCAGGGTCCGAGGTATTC
miR-15b	Reverse transcription	stem-loop RT	CTCAACTGGTGTCGTGGAGTCGGCAATTCAGTTGAGTGTAAACC
	real time PCR	forward	ACACTCCAGCTGGGTAGCAGCACATCATGG
		reverse	Universalreverse primer as above
miR-15b	mimics	sense	UAGCAGCACAUCAUGGUUUACA
		Anti-sence	UAAACCAUGAUGUGCUGCUAUU
Negative control	mimics	sense	UUCUCCGAACGUGUCACGUTT
		Anti-sence	ACGUGACACGUUCGGAGAATT
HBVRNA	real time PCR	forward	CCGTCTGTGCCTTCTCATCT
		reverse	TAATCTCCTCCCCCAACTCC
miR-15b sponge	pCAGGs-15b sponges cloning	sense	GCCGGATCCTGTAAACCATCATTGCTGCTACTTCTGTAAACCATCATTGCTGCTAC
		Anti-sence	GCCAAGCTTAGATCTTAGCAGCAATGATGGTTTACAGAAGTAGCAGCAATGATGG
miR-15b Gsensor	miR-15b Gsensor	sense	AATTCTGTAAACCATGATGTGCTGCTAC
		Anti-sence	TCGAGTAGCAGCACATCATGGTTTACAG
Enhancer I	pGL3- Enhancer I	forward	GGTACCGGGAACGTTGCCACAGGATC

	construction	reverse	AAGCTTGACCGGCTGCGAGCAAAAC
S1p promoter	pGL3- S1p	forward	GGTACCCTTGCCTTACTTTTGGAAG
		reverse	AAGCTTAAGAATATGGTGACCCGC
S2 p promoter	pGL3- S2p	forward	GGTACCCAAACAATCCAGATTGGGAC
		reverse	AAGCTTACTGCCGATTGGTGGAGG
Cp promoter	pGL3-Cp	forward	GGTACCGCCCATCAGATCCTGCCCAAG
		reverse	AAGCTTGAAAAAGTTGCATGGTG
HBx	HBx labeled for southern blot	forward	ATGGCTGCTAGGCTGTGCTGCCAAC
		reverse	GGCAGAGGTGAAAAAGTTGCATGGTG
HNF1 α	HNF1 α-3’UTR cloning	forward	GAATTCTAACCACGGCACCTGGGCCCTG
		reverse	CTCGAGAAACGTGCCACTCGCCCCGGCTG
HNF1 α	real time PCR	forward	GGAGGAGCGAGAGACGCTAGT
		reverse	CACCCCTCTCTGGATGCATT
β-acin	real time PCR	forward	CGTGGACATCCGCAAAGAC
		reverse	CTCAGGAGGAGCAATGATCTTGAT
HNF1 α	pcDNA3.1- HNF1 α cloning	forward	GCCACCATGGTTTCTAAACTGAGCCAGC
		reverse	TTACTGGGAGGAAGAGGCCATCTG
si HNF1 α	pSilencer- HNF1 α construction	sense	GATCCGAAGAAGCCTTCCGGCACATTCAAGAGATGTGCCGGAAG GCTTCTTCTTTTTTGGAAA
		Anti-sence	AGCTTTTCCAAAAAAGAAGAAGCCTTCCGGCACATCTCTTGAATG TGCCGGAAGGCTTCTTCG
Mutation in HNF1α binding sites	pGL3- Enhancer I-M	forward	ACAGCAAAAGCCCGAGCGACCTTCGGTACCGGGAACGTTGCCAC AGGATC
		reverse	GTTTTGCTCGCAGCCGGTCAAGCTTCGGCTAAGGCACGCGCCACT TTT

		mutant	GTTTTAGAAAACTTCCTACCGGCAGGCCTATTGATTGG
Mutation in miR-15b binding sites	HNF1α-3’UTR -Mutant	forward	ACAGCAAAAGCCCGAGCGACCTTCGAATTCTAACCACGGCACCTGGGCCCTG
		reverse	CAGCCGGGGCGAGTGGCACGTTCTCGAGCGGCTAAGGCACGCGCCACTTTT
		mutant	CACATCCCTGGGCCTCCACCTGAGAACCTGGCCTTC

Supplementary figure legends

Figure S1. Ectopic miR-15b expression promotes HBV replication and expression.

Intracellular HBV DNA replication intermediates were detected by Southern blot and qRT-PCR in HepG2.2.15 cells (A) transfected with 40 nM miR-15b mimics/NC, or Huh-7 cells (C) transfected with pHBV1.2 plus 20 nM miR-15b mimics/NC. The positions of relaxed circular (RC), double-stranded linear (DL), and single-stranded (SS) DNAs were indicated in the southern blot. (B) Proliferation of Huh-7 cells transfected with miR-15b mimics/NC. Cell proliferation was evaluated by MTT assay.

Figure S2. Evaluation of pCAGGs-15b transduction efficiency

Three days after injected with pCAGGS-15b or pCAGGs, the relative expression of miR-15b (A) and its target (HNF1α) (B) in C57BL/6 mice liver were detected by qRT-PCR.

Figure S3. The effect of HNF1α on HBV Enhancer I

(A) Luciferase reporter assay was performed 48 h after cotransfection with the wild-type or the mutant HBV Enhancer I and pSilencer-HNF1α or pSilencer. (B) The relative activity of wild-type or mutant Enhancer I in Huh-7 cells.

Figure S4. Ectopic miR-15b expression decreases HNF1α mRNA and protein expression in HepG2.2.15 cells.

The expression of HNF1α mRNA (upper) and HNF1α protein (lower) was analyzed in HepG2.2.15 cells transfected with 40 nM miR-15b mimics/NC.
